# Medication safety in community pharmacy: a qualitative study of the sociotechnical context

**DOI:** 10.1186/1472-6963-9-158

**Published:** 2009-09-07

**Authors:** Denham L Phipps, Peter R Noyce, Dianne Parker, Darren M Ashcroft

**Affiliations:** 1 School of Pharmacy and Pharmaceutical Sciences, University of Manchester, Manchester, UK; 2 Manchester Business School, University of Manchester, Manchester, UK; 3 Safety Culture Associates Ltd, Buckley, Flintshire, UK

## Abstract

**Background:**

While much research has been conducted on medication safety, few of these studies have addressed primary care, despite the high volume of prescribing and dispensing of medicines that occurs in this setting. Those studies that have examined primary care dispensing emphasised the need to understand the role of sociotechnical factors (that is, the interactions between people, tasks, equipment and organisational structures) in promoting or preventing medication incidents. The aim of this study was to identify sociotechnical factors that community pharmacy staff encounter in practice, and suggest how these factors might impact on medication safety.

**Methods:**

Sixty-seven practitioners, working in the North West of England, took part in ten focus groups on risk management in community pharmacy. The data obtained from these groups was subjected to a qualitative analysis to identify recurrent themes pertaining to sociotechnical aspects of medication safety.

**Results:**

The findings indicated several characteristics of participants' work settings that were potentially related to medication safety. These were broadly classified as relationships involving the pharmacist, demands on the pharmacist and management and governance of pharmacists.

**Conclusion:**

It is recommended that the issues raised in this study be considered in future work examining medication safety in primary care.

## Background

Medication safety has long been recognised as a key issue within the broader patient safety agenda [1-3]. A number of studies have shown that medication error is relatively common and identified a range of contributory factors occurring at the individual (for example, fatigue and training) and organisational (for example, staffing, organisational climate and system design) levels of analysis [4-6]. Much of this research has been conducted in secondary care settings, with relatively few studies taking place in primary care, and fewer still focusing on the dispensing of medicines in community pharmacy. However, a large proportion of medication prescribing and dispensing occurs in primary care settings [3]. The British National Health Service [7] reports that approximately 11,000 community pharmacies existed in England and Wales, dispensing approximately 785 million prescription items each year. While there is no population-level estimate for the incidence of adverse events in primary care dispensing, a prospective self-report study in a sample of 35 British community pharmacies found an incidence rate of 22 near misses and four errors for every 10,000 items dispensed [8]. A similar error rate (0.26%) was found in an observational study of a single pharmacy in the United States [9]. In both cases, errors were attributed to a range of factors, including misidentification of drugs, workload, distractions and dispensing against product labels created during the process rather than against the prescription itself. Meanwhile, Peterson, Wu & Bergin [10] note concerns among Australian community pharmacists that an increase in workload is creating more opportunities for dispensing errors to occur, and Szeinbach, Seoane-Vazquez, Parekh & Herderick [11] found a relationship between volume of prescriptions and frequency of dispensing errors in a self-report survey of US community pharmacists.

These studies highlight the need to address what have been termed "sociotechnical" factors in medication safety. Sociotechnical factors are those concerning the relationship between the technical, psychological and social elements of a work system [12]. Their relevance to community pharmacy is illustrated by Ashcroft, Morecroft, Parker & Noyce [13], who note the influence of both technical elements (such as workload and staffing) and social elements (attitudes towards incident reporting and organisational learning) on the proliferation of medication incidents. During their investigation, Ashcroft et al. collected qualitative data from a sample of community pharmacy staff. Their analysis of this data, though, focused only on reporting and learning from medication incidents, their aim being to develop a safety culture assessment tool [13,14]. The aim of the present study is to extend Ashcroft et al.'s initial work by identifying sociotechnical factors that participants felt were related to medication safety in general - that is, the causation, detection and prevention of medication incidents as well as their reporting.

## Method

The authors carried out a secondary analysis of the qualitative data collected for Ashcroft et al.'s [13] study; the original aim of which was to explore participants' views about risk management culture in community pharmacy. The data was collected from ten focus groups conducted in the North West of England between December 2003 and April 2004. These focus groups involved a total of 67 participants, all working in community pharmacy. Participants were recruited on a purposive basis, having taken part in a previous questionnaire study by the authors and consented to be contacted about further research. The participants represented a range of roles and locations across the region, as shown in Table 1. Ethical approval for the research was granted by the University of Manchester Senate Committee.

**Table 1:** Composition of focus groups

**Group**	**Pharmacists**			**Pre-registration pharmacists**	**Support staff**
			
	**Owner**	**Employed**	**Locum**		
1		1	6		
2		2	4		
3			7	1	
4		4			
5				9	
6		1	3		
7					4
8	2	3	1		1
9			10		
10	8				

Total N	10	11	31	10	5
% of sample	14.9	16.5	46.3	14.9	7.4

Note: "Locum" is the UK term for sessional pharmacists. "Pre-registration" is the UK term for trainee pharmacists. "Support staff" is a collective term for counter assistants and non-pharmacist dispensers.

Each focus group lasted for approximately two hours and was moderated by an experienced qualitative researcher. The topic guide was semi-structured, and included the following themes:

• What are the attitudes and views of pharmacists and support staff about patient safety in community pharmacy?

• What is the prevailing culture with respect to patient safety in community pharmacy?

• How does the prevailing culture affect how incidents are handled?

• What types of incidents are reported/not reported in community pharmacy?

• What factors facilitate and inhibit reporting?

As well as these themes, other discussion topics were allowed to emerge spontaneously during each focus group. All focus group discussions were tape recorded and fully transcribed for subsequent analysis.

Two researchers (DLP and DMA) identified themes relevant to the sociotechnical aspects of community pharmacy using template analysis [15,16]. Template analysis is an inductive process that involves the analyst reading through the data and creating a "template" consisting of the general themes that emerge from this reading. The template is then modified and extended through successive readings until it provides sufficient coverage of the data. In the current study, the researchers began by reading the entire dataset and identifying the general issues that participants raised in relation to medication safety. These issues became the superordinate themes. They were used as a framework to categorise the data from each focus group, following which each theme was developed by identifying subordinate themes in the data assigned to it. Version 7 of NVivo [17] was used to document the analysis, which continued until the analysts had identified as many themes as possible from the data. As DLP and DMA worked separately to identify superordinate themes, reliability of the analysis was established by comparing the two set of themes for consistency, with the final template incorporating both sets; this being a common approach to establishing reliability in qualitative studies that use a realist epistemological framework [18]. The final template, consisting of the superordinate and subordinate themes, was reviewed by PRN and DP to confirm the relevance of the themes to medication safety.

## Results and discussion

Across all of the focus groups, the factors thought to affect medication safety were summarised using three superordinate themes: relationships involving the pharmacist; demands on the pharmacist; managing pharmacy work. These themes, and their subordinate themes, are listed in Table 2.

**Table 2:** Sociotechnical factors related to medication safety

**General themes**	**Subordinate themes**
*(i) Relationships involving the pharmacist:*	
Peers	Group norms
	Involvement of locums
Other health care professionals	Collaboration with prescribers
	Pharmacist involvement in governance
Patients/customers	Customer demands
	Trust in pharmacist
	Patient as final safety barrier
	Informing patients about safety issues
	
*(ii) Demands on the pharmacist:*	
Commercial	Profitability vs safety
	Financial dependencies
	Budgetary constraints
Corporate	Approach to governance
	Organisational culture
	Hierarchy and protection
Legal and regulatory	Legal and regulatory sanctions
	Following the law vs meeting demands
	Support from regulator
	Regulator enforcing standards
	
*(iii) Management and governance:*	
Blame culture vs learning culture	Allocating/accepting blame
	Learning from experience
	Openness and trust
	Being the target of blame
Formal vs informal processes	Monitoring and audit
	Reporting and feedback
	Trust and engagement in governance
	Communities of practice
Protocols	Quality assurance
	Individuality and professional autonomy
	Credibility and practicality
	Doing what's best for the patient
Work design	Human-computer interaction
	Workspace
	Automation

### Relationships involving the pharmacist

The first theme - relationships involving the pharmacist - reflected the interdependencies and interactions between the pharmacist and other people. These include peers, other health care professionals outside the pharmacy, and service users.

#### Peers

Pharmacists highlighted the role of their professional peers in maintaining safe practice. As with other professional groups, collective norms of practice may emerge amongst pharmacists. However, while some of these norms may encourage safe practice, others may discourage it.

[We] were taught, certainly in my era, at College, you did not make mistakes, you covered them up, that was the history. I had a boss who I could have killed because he did make mistakes but he refused to admit it. [...] [I] went in to the dispenser and said "Look, ignore him, we all make mistakes, we check each other" [Locum pharmacist, Group 9]

The effect of professional norms on safety-related behaviour has been noted in previous studies; for example, anaesthetists' approaches to the use of guidelines are influenced to some extent by how they expect their peers to act [19,20]. Hence one of the ways to achieve medication safety is to encourage pharmacists to develop group norms that support safe behaviour.

Other comments made by participants referred to perceived differences between agency-supplied (locum) pharmacists and permanent (employed) pharmacists with regard to involvement in pharmacy activities.

I [once] had an issue with methadone, [...] I did not agree with what the pharmacists had done the previous week and they'd done nothing about it all week when they had time to sort it out and then they didn't even tell me in advance of me going, I walk in on the Saturday and get stuck with the real issue, do you give it, don't you give it. And you're dealing with something that's quite, you know, can change that person quite a lot, and you're thinking, "Well, where do you stand?" [Locum pharmacist, Group 3]

The locum pharmacist talks of his disconnection from the day-to-day activity of the pharmacy; this can lead to pharmacists (whether employed full-time, part-time or locum) being confronted with an ongoing or complex situation of which they have only partial knowledge. This may become more of a problem as pharmacies extend their opening hours, increasing the need for shift handovers between both pharmacists and support staff [21]. In any case, there is perhaps a need for more documentation of cases for the purposes of information sharing.

#### Other health care professionals

Some participants commented on the relationship between pharmacist and prescriber. In particular, issues related to the partnership working that occurs between the two, for example the pharmacist agreeing to dispense an incomplete prescription on the understanding that the prescriber will issue an amended version later. However, there was also a fear on the part of pharmacists that they would be blamed for medication incidents that actually originated with the prescriber.

When a prescription comes into us we're relying on the fact that the GP has made the correct diagnosis and has prescribed a dose that is safe for that patient. [...] So [...] you're already working, even with prescription medicines, from half the song sheet, aren't you? [Pharmacy owner, Group 10]

I would send [an incomplete prescription] back even if I knew the doctor. If you accept it the first time and you keep accepting it, [...] it'll happen again. [...] I'm just not going to accept the responsibility for the doctor's mistake. [Locum pharmacist, Group 1]

These excerpts point to a tension within the pharmacist-prescriber relationship. On the one hand, pharmacists are keen to protect their dispensing from substandard prescribing, particularly if they may be held culpable for any problems that result. On the other hand, pharmacists are dependent on prescribers for their business, and so may be reluctant to disrupt their working relationship. Bradley et al. [22] have noted that trust in each others' capabilities is fundamental to collaborative working between pharmacists and GPs. It could be hypothesised that the extent to which both parties are willing to collaborate over safety-related issues (for example by holding each other mutually accountable for adhering to prescribing procedures) is determined by the level of interpersonal or professional trust that they have in each other.

#### Patients/customers

Several participants implied a role for the patient as a safety barrier, as there were situations in which the patient forestalled a potential adverse event by identifying an incorrectly dispensed medicine. However, while some participants felt that it would be useful to involve the public in safety issues, others were reluctant to do so because of a fear that this would erode patients' trust in the pharmacy.

I [once] called the name, didn't ask the address, spent ten minutes counselling [the patient] on how to use an inhaler and [he] came back and said "This is the wrong thing. I was expecting tablets". [...] That was my mistake, [but] that goes to show you how much he was listening [Locum pharmacist, Group 1]

We want to [...] expand our role, and if the patients aren't gonna have any confidence in us in like medication reviews and prescribing, if they think we're not as competent [...]. That's my concern, I just don't want them to lose confidence in us because I want pharmacists to do those extended roles [Employed pharmacist, Group 4]

It is interesting to consider where the power lies in the pharmacist-customer relationship. From one point of view, the pharmacist has a basis for power, as it is he or she rather than the customer who has insight about performance and safety issues in the pharmacy, as well as being the one who is responsible for managing any interactions with the customer. However, some of the comments made suggest that the customer has some influence over the pharmacist; the latter perceives pressure to turn around medicines quickly for the customer. This may be of particular relevance to medication safety given that one source of high workload could be the perceived or actual productivity demands from customers, as well as interruptions to the pharmacist [10].

I think we've got to get away from the idea that a good pharmacist in the view of the public is one who gets the medication out quickly. [...] They just assume that it's going to be correct but they don't rank the actual quality of the dispensing in any of it, they put speed at the top [Locum pharmacist, Group 9]

I've got an open plan pharmacy, and I've got people right over the top and talk about interruptions. [...] It takes your whole attention away. [...] The patient can be the biggest distraction you have [Pharmacy owner, Group 10]

Incidentally, there is some debate in the literature about how, and how much, patients should be formally involved in medication checking. Lyons [23] argues that, from a safety engineering perspective, it would be difficult to ensure that patients provided a consistent and reliable safety check, given the diversity of patients and a possible unpreparedness to take responsibility for safety of their treatment. However, Entwistle [24] argues that, from a medical ethics perspective, it would be beneficial to involve patients in the final check, even if it is impractical to place complete reliance on them to do the check themselves, as this can enhance carer-patient communication and provide an opportunity for patient education. The extent to which patients should be involved in medicine checking in community pharmacy, and how much pharmacists want them to be involved, is a question that may warrant further investigation.

### Demands on the pharmacist

As well as interacting with different people and groups, the community pharmacist operates within a number of constraints. These arise both from business needs and from the legal and regulatory context of practice.

#### Commercial

In the UK, community pharmacy differs from many other healthcare services in that it operates as a private business, rather than being government owned. This means that a key concern for community pharmacists is the cost of their services and the financial impact of any decisions made in the course of their work. Commercial concerns could potentially influence the attitude of pharmacists to service provision.

We're gonna be [...] competing for money [from the Primary Care Trust] cos they're gonna have seventy five percent of the budget and [...] we're gonna want their services, so it might prevent us from being honest [with them] about our mistakes and errors [Employed pharmacist, Group 4]

It's very hard to make it work when you've got [...] whistle-blowers in two different sorts of organisation. Like non-profit organisations like nurses and GPs, and pharmacists in profit organisations, we're supposed to whistle-blow on GPs if we see bad prescribing, but to the detriment of our business? [Pharmacist, Group 8]

Staff is the single most expensive item in any business, whether it be pharmacy or anywhere else, and all the firms [...] are under pressure from their shareholders to make more profit, and the easiest and quickest way to do that is not increasing turnover, it's to cut down the staff [Locum pharmacist, Group 9]

The first two quotes concern the integration between community pharmacies and the not-for-profit sections of the health service. The pharmacist in Group 4 expresses a reluctance to report errors to the body that is responsible for funding the pharmacy, while the pharmacist in Group 8 suggests that community pharmacists have more to lose than do other healthcare providers by reporting others' bad practice. Hence, the relationship between pharmacists and the safety management functions of the health commissioning organisation may be influenced by the pharmacists' need to protect their business. The third comment refers to a perceived conflict between the need to meet commercial targets and the need to ensure adequate resources - in this case, staff. Shortage of staff, though, may have adverse consequences for safety in the face of increasing workload in pharmacies [4,10].

#### Corporate

In the UK, community pharmacies come in a number of forms - some operate as independent stores; others as small chains consisting of several stores; yet others as larger regional or national chains, often integrated with a general department store or supermarket. As organisations in general vary in terms of their culture and hence operating practices [25], so do these different types of pharmacy. In general, larger chains were seen as having a more centralised approach, in which pharmacists at the various chain locations were expected to take direction from senior managers based in a headquarters.

I think the protocols that [chains] have as well tend to be stricter [than in independents], and they won't let you bend from the protocols [Employed pharmacist, Group 6]

No, because they've got so many branches to cover, they've got to put it down as a must do, rather than a, well, we'll get round it type of thing [Locum pharmacist, Group 6]

The advantage of notifying the Head Office [of a chain] is that they then cascade the information to everybody so that every store can then separate the Xalacom and Xalitan in the fridge so that it doesn't happen, so you're actually avoiding the error ever happening [Locum pharmacist, Group 9]

I think you tend to get more demoralised staff in a company and more negative on feedback and communication to a well run independent sometimes, because I think they're sort of all put into a block and they can be boxed if you're not careful in a company [Locum pharmacist, Group 1]

As the first two quotes suggest, the centralised approach of pharmacy chains carries some benefits for safety management. First, by establishing a managerial hierarchy and formally defining standards of practice, it provides a form of quality assurance. Second, the organisation may have mechanisms for sharing resources and knowledge. However, the third quote surmises that differences in morale and communication between staff may be found across the different types of pharmacy. Whether this is the case is not clear from the data in this study; however, Ashcroft & Parker [26] suggest that staff commitment and communication (as well as resourcing) are amongst the factors associated with safety in pharmacies. Hence, it would be of value to evaluate the effect of organisational structures and practices on safety.

#### Legal and regulatory

Pharmacy practice in the United Kingdom is governed both by legislation such as the Medicines Act, and by the regulations of the pharmacy professional body, which at the time of writing is the Royal Pharmaceutical Society of Great Britain. Hence there are statutory requirements by which pharmacists need to abide. As autonomous practitioners they are very sensitive to the risk of disciplinary action or litigation should a patient be harmed. This emerged as a key driver for safe practice.

Getting struck off the register, the thought of that, and as a professional as well, not being able to practice. So it's all kinds, you have a lot to lose as a pharmacist [Employed pharmacist, Group 4]

It's all about going to court, isn't it, that's the last thing anybody wants [Employed pharmacist, Group 4]

*You get somebody coming back and say, [...] "I was owed five bendrofluazide tablets." And there's absolutely no record [...], somebody's [must have] made a mistake at this end [...], so [...] you give it out. Now, technically that's going against the law [...] [but] at the end of the day there's a little bit of a grey area [Locum pharmacist, Group 6]*.

The pharmaceutical inspector [...] had a woman who was on fentanyl patches [...]. The pharmacist actually lent her a pack of five, on the understanding that the [...] surgery [was] going to write the prescription. [...] [However, the] surgery would then not provide a prescription. [...] [The inspector] said there was no way I was going to jump on him for giving those five patches which she'd been having for [...] seven or eight months, because the pharmacist had done the right thing by the patient [Locum pharmacist, Group 6]

There was a general disinclination on the part of participants to incur the wrath of the law or the regulator. However, some participants described situations in which pharmacists may feel they should "bend" the law, suggesting a direct conflict between doing "the right thing" and staying within legal boundaries. Interestingly, the last quote suggests that the regulator may also play a role in arbitrating between the two imperatives. Benson, Cribb and Barber's study of pharmacy practitioners [27] also described situations in which ethical responsibilities and legal responsibilities were felt by the respondent to be in conflict; however, the relative frequency of such situations is not fully clear. Another issue to consider is which course of action (legal or ethical) is felt by pharmacists to be most closely aligned with safe practice.

A further potential role that participants identified for the regulator is to negotiate with pharmacy employers and drug manufacturers in order to ensure adequate resources for safe practice.

We've been calling for [manufacturers to change] the packaging and colour [coding] and all the rest of it, we've been calling for this for years, and nothing's happening because [...] no individual body has got sufficient clout, so it needs to go to one central body to actually be able to say "Enough is enough" [Locum pharmacist, group 9]

### Management and governance

In order to manage the work of pharmacists, both their employers and their professional regulator may make use of a range of methods. These include governance processes such as incident reporting, regulating practice through the use of protocols, and the physical design of workplaces and tools. All of these can influence the work of pharmacists as shown in the following examples.

#### Blame culture vs learning culture

Some of the participants recognised the benefits of developing a culture in which incidents were openly discussed and lessons shared and acted upon. However, many participants felt that a barrier to adopting such practices is a tendency to attribute blame to individuals unnecessarily.

One of the issues [...] is getting rid of this blame culture. Although we're trying to instil [that] it doesn't really matter who did it, [...] let's move on and learn from it, people are still very hung up on who actually committed the error [Locum pharmacist, Group 9]

Even in our branch they say it doesn't matter if you've done wrong, [...] just write your name down here but nobody likes their name put down. [...] You get a bit paranoid, you think [...] it's just your name there and you think you must be absolute rubbish at your job [Pre-registration pharmacist, Group 5]

The effect of blame culture on safety management in healthcare settings has been discussed in depth in previous literature [28]. Here, it will suffice to say that the comments made by participants in this study are consistent with the findings of previous studies; it would appear that sensitivities about being seen to blame colleagues, or being the subject of blame oneself, may provide a barrier to exploring and learning from adverse events in pharmacy practice [29].

#### Formal vs informal approaches

While there are usually formal governance processes - mainly based on reporting and audit - some participants alluded to the alternative use of informal approaches. For example, a pharmacy store manager may, having found out about a dispensing error, discuss it with the people involved in the first instance, only resorting to formal reporting if the matter cannot be resolved informally. Also, some participants noted that their level of trust in governance processes depends on who is administering them.

Newly qualified [...], couple of days in, made a mistake, and I told them and we talked about it, but I didn't report it to head office, cos I knew that they were devastated [...] I gotta think about this person and they would probably not wanna be a pharmacist any more. [...] My thought [was that] they really learnt from this, and I would be surprised if they did it again [Pharmacist, Group 2]

If your reports are gonna go "Look, this pharmacy's got this much near misses, ooh, it's a black-listed pharmacy, this one." But if they're gonna [...] recognise that you've got these amount of near misses or whatever, this is what you could do, this what you could do to improve. [...] Is it gonna be against you or for you in that respect? [Pharmacist, Group 8]

There's no point in being proactive to a system or management or a body which is itself being reactive and disciplinary, because that defeats the point of you being proactive in the first place [Pharmacy owner, Group 10]

The data suggested that pharmacists will engage with any governance system - whether formal or informal - to the extent that it supports development of practice rather than sanctioning individuals or sites. This may reflect the presence of a pharmacist community of practice - that is, a social group that fosters collective learning and norms of practice [30]. Communities of practice tend to be informal and peer-led, rather than management-led, and so healthcare professionals may prefer to engage with them than with formal governance structures [31]. The implication is that those responsible for managing pharmacists should be aware of the existence of communities of practice and work with them, rather than against them, for mutual benefit.

#### Protocols

Protocols were recognised to be important in principle, as they set minimum standards for practice. However, some participants felt that an over-reliance on protocols ran counter to their belief in being autonomous professionals.

The protocols are set up because that gives the best chance of success and the best patient outcome, now you can still have a good patient outcome even though the protocol's not been adhered to, but [that could be by] luck [or] whatever [Pharmacy owner, Group 10]

I know other pharmacists who [...] definitely stick to the rules, no matter what, and are not gonna bend 'em. Then some people who kind of just squeeze past them. So it does depend on the person [Pharmacist, Group 2]

If you've got a patient who's at risk and if you're doing something or not doing something, then I would ignore [the protocol] and do what I thought was the professional way, would be the best thing to do [Pre-registration pharmacist, Group 5]

I sometimes think they're not very helpful for patients. If you've got a protocol and you've got to do it a certain way, but then you can't, say a checking one [...] do you break it for the benefit of the patient, even though you know you shouldn't? [Pharmacist, Group 2]

The participants explained that there can sometimes be a conflict between what a protocol dictates that the pharmacist should do and what the pharmacist judges to be the right thing to do in the situation. It may be the case that a number of factors play into the decision whether or not to follow a protocol - for example, the nature of the protocol, the experience of the pharmacist and the nature of the situation at hand [19,20]. Nevertheless, deviations from protocols have been implicated in adverse events in other healthcare domains [32], and so there is a need to consider the relevance of pharmacy protocols to medication safety.

#### Work design

A key feature of the work environment that was reported to influence pharmacists' work is its physical manifestation - that is, the layout of the workspace and the equipment that is found within it. New technologies - such as decision support or automation - may change the nature of the pharmacist's task activity and demands.

If you've got [...] [a] layout [in] which you're literally right behind the sales counter you can eavesdrop on pretty much any conversation, if you've got somebody at the far end of the shop ringing a bell every time [they're selling] a bottle of Calpol, then you're less able to intervene [Pharmacy owner, Group 10]

The "goldfish bowl" dispensary is not a good thing [Locum pharmacist, Group 9]

You've got to have a tidy dispensary or things do get muddled up and then when you're working fast you're just leaping around, grabbing things off the shelf, and unless things have been put in the correct place it's so easy [to make a mistake] [Locum pharmacist, Group 9]

If you start looking at the dispensing process as two stages, there's the mechanical process and there's the clinical process. [...] [If] you divest the two and use [automation] to take away some of the human error [...] while still allowing the clinical input [...] you stop worrying so much about "Am I gonna make errors that are gonna go out to the patient?" [...] That becomes less of an issue and you start thinking more about "Hang on a minute, is what's being dispensed in the best interest of the patient anyway?" [Pharmacy owner, Group 10]

Szeinbach et al. [11] and Rapport, Doel & Jerzembek [33] have observed that the topography of community pharmacists' work areas can either facilitate or interfere with their practice. For example, Rapport et al. note that a well-organised workspace engenders a sense of professionalism and reduces the likelihood of dispensing error, while layouts that make pharmacists too accessible to customers can invite ill-timed interruptions to consultations or dispensing. Respondents to Szeinbach et al.'s survey suggested that the configuration of the pharmacy (that is, its shape and the presence of a drive-through window) could affect the efficiency and accuracy of dispensing; automation, though, was generally felt not to have a negative impact on efficiency and accuracy. As suggested by the quotes here, participants in the current study held similar views about layout to those expressed in these previous studies. The fourth quote refers to the impact of automated dispensing; the pharmacist's role - much like other operators working with closed-loop control systems, such as pilots and anaesthetists - becomes one of monitor and decision-maker rather than hands-on assembler. This change may or may not be welcome to particular pharmacists.

### Study design: strengths and weaknesses

The use of semi-structured focus groups provided the researchers with a large volume of rich data about the experiences of community pharmacy staff. The open-ended nature of the data collection allowed the participants to raise other issues that might not have been considered by the researchers. However, the findings may be limited in that the sample was confined to community pharmacy staff working in the North West of England. While the researchers expect that the findings apply to pharmacies elsewhere in the United Kingdom, if not internationally, it is possible that the study has highlighted some issues that are specific to the geographical location in which it was conducted. The sample consisted predominantly of locum pharmacists. While a 2002 workforce survey also indicated a high proportion of locums in comparison to other types of pharmacists [34], it is possible that the views of locums have been given undue predominance in the current study. Support staff and technicians had relatively little representation in the sample. Also, there is a time lag of five years between the focus groups and the publication of the current paper. While the authors would suggest that the issues raised in this paper remain of relevance, it is acknowledged that they predate more recent developments in pharmacy practice such as extended services (such as supplementary prescribing) and registered pharmacy technicians (who, unlike many support staff at the time of the present study, have nationally-recognised formal training and professional accreditation), as well as the forthcoming transfer of pharmacist regulation to an independent council. Any of these developments may change the views of pharmacists about sociotechnical influences on safety; alternatively, the changes may be viewed from the perspectives described here. In any case, what cannot be inferred from this study is the predictive or causative relationship between any of the factors indicated and measures of quality or safety. Such a link should ideally be a focus for future research. One way of studying this may be to use the factors discussed here as the basis for a questionnaire, the data from which could be subjected to factor and regression analysis.

## Conclusion

While medication safety might be viewed in terms of the dispensing process itself, the focus group data from community pharmacy staff indicate various social and organisational factors that also have a potential impact. Some of these issues are common to both hospital and community dispensing. However, others are peculiar to community pharmacy, in particular the strongly commercial nature of services in this setting. Either way, it is important to examine their origin, nature and influence on safety.

## Competing interests

The authors declare that they have no competing interests.

## Authors' contributions

DMA and DP contributed to the design and data collection of the original study. DLP and DMA carried out the data analysis for the current study. PRN and DP reviewed the findings of this analysis. The manuscript was written by DLP and approved by the co-authors.

## Pre-publication history

The pre-publication history for this paper can be accessed here:


